# Intelligence

**Published:** 1809-09

**Authors:** 


### INTELLIGENCE.

ROYAL COLLEGE OF SURGEONS.

The Jacksonian Prize-Subject for the year 1810, for the premium of 10*l.* to the Author of the best Dissertation on a practical Subject in Surgery, is the BITE of a RABID ANIMAL. Candidates must be Members of the College, not on the Court of Assistants. The Dissertations must be in English; and the number and importance of facts will be considered the chief points of excellence in the compositions. Each dissertation must be distinguished by a motto or device; but no dissertation, motto or device, must be in the hand-writing of the author. Every dissertation must be accompanied by a paper, sealed up, (not with the seal of the author) containing the name and residence of the author; and having, on the outside a motto or device, (not in his own hand-writing) corresponding with the motto or device on the dissertation. Dissertations must be addressed to the Secretary, and delivered at the College before Christmas-Day, 1810. The adjudication will be in the month of April, 1811. The prise-dissertation will be preserved in the Library of the College. The unapproved dissertations, with their correspondent unsealed papers, will be returned, upon authenticated applications. Every unapproved dissertation, which shall remain three years unclaimed, and its correspondent paper, unopened, will be burnt in the presence of the Jacksonian Committee.—The Prize-subject for the present year, 1809, is Fractures of the Bones of the Trunk, and the Treatment of them; dissertations upon which must be delivered before Christmas-Day next.

---

DURING the last month an Ophthalmia has prevailed so generally, that it may be considered as approaching to an epidemic in some districts. The young have been more frequently attacked than adults, and in some places it has appeared to spread by contagion; but most probably this has not been the case. It has been thought to prevail earlier, and more extensively about the west end of the metropolis, and places on the west of it, especially Chelsea. The number of soldiers who have been affected with the Egyptian Ophthalmia, which is contagious, and who have been sent to the York Hospital at Chelsea, has probably given rise to the opinion of its contagious nature. But this disease is in all respects unlike that, being only a moderate inflammation of the adnata, and, in most cases, requiring no other remedy than the frequent application of cold water, and seldom continuing more than three or four days.——When any thing like an epidemic makes its appearance, practitioners seek for the cause of it either in contagion, particular winds, or weather, but neither of these appears to have any share in producing the above complaint.

The hooping cough having been more general than for some years, after a pretty long cessation, affords a fair opportunity for judging whether that disease is to be considered an endemic, or a common contagion. We shall be glad of any communications on that subject.

Captain Dick, of the 21st Light Dragoons, having witnessed the good effect of the Arctopus echinatus of Linnæus, in his regiment at the Cape of Good Hope, has, much to his honour, brought over a sufficient quantity in substance and in tincture, to make ample experiments of its virtue. The charge is intrusted to Dr. Adams, who will make as early a report as such experiments will admit.

A few years since, Messrs. Fourcroy and Vauquelin remarked, that a concrete sugar, or manna, exuded from the receptacle of the flowers of the pontic dwarf rosebay (*Rhododendron ponticum*). M. Bosc has recently observed it afresh, and presented to the National Institute some grains of this substance, collected from the receptacle of the fruit, several of which were about $\frac{5}{12}$ of a line in diameter. Their taste and appearance do not differ perceptibly from the purest sugar-candy; but it is necessary to be upon our guard against this appearance, on account of the deleterious properties suspected in the plant. This manna, according to M. Bosc, is dissolved during the night by the moisture of the atmosphere, melted in the day by the heat of the sun, and does not exude from plants of a vigorous vegetation. These are the reasons why it is so seldom seen. Plants growing in pots, and sheltered from the dew, as well as from the sun, are most likely to furnish it. The grains above mentioned, were collected from a plant, in which all these circumstances united.

---

### UNIVERSITY OF GLASGOW.

The Medical Lectures in the University of Glasgow, will begin on Wednesday, the 1st of November, at the following hours.

Dietetics, Materia Medica, and Pharmacy, by Dr. Millar, at Ten o'Clock in the Forenoon.

Midwifery, by Mr. Towers, at Eleven.

Theory and Practice of Physic, by Dr. Freer, at Twelve.

Anatomy and Surgery, by Dr. Jeffray, at Two o'Clock in the Afternoon.

Chemistry and Chemical Pharmacy, by Dr. Cleghorn, at Seven.

Clinical Lectures on the cases of patients in the Royal Infirmary, by Dr. Freer and Dr. Cleghorn. The first Lecture by Dr. Freer, on Tuesday Evening the 14th of Nov. at Six o'Clock.

Dr. Brown will commence his Lectures on Botany, about the beginning of May next.

The following gentlemen have obtained the degree of Doctor i Medicine, from this University, within the last twelve months.

Mr. John Monteath, from Scotland.
Mr. Robert Muir, from Scotland.
Mr. William Young, from Scotland.

Mr.

Mr. ROBERT PERRY, from Scotland.
Mr. ROBERT NEILSON, from Scotland.
Mr. FRANCIS CAVENAGH, from Ireland.
Mr. WILLIAM LANGMORE, from England.
Mr. JOHN CROZIER, from Ireland.
Mr. MICHAEL DILLON, from Ireland.
Mr. JOHN WHITTAM, from England.

---

St. THOMAS'S AND GUY'S HOSPITALS.

The Winter Courses of Lectures at these adjoining Hospitals, will commence the beginning of October, viz.

*At St. Thomas's.*

Anatomy and the Operations of Surgery, by Mr. CLINE and Mr. COOPER. Principles and Practice of Surgery, by Mr. COOPER.

Practice of Medicine, by Dr. BABINGTON and Dr. CURRY.—Chemistry, by Dr. BABINGTON, Dr. MARCET, and Mr. ALLEN.—Experimental Philosophy, by Mr. ALLEN.—Theory of Medicine, and Materia Medica, by Dr. CURRY and Dr. CHOLMELEY.—Midwifery, and Diseases of Women and Children, by Dr. HAIGHTON.—Physiology, or Laws of the Animal Œconomy, by Dr. HAIGHTON.—Structure and Diseases of the Teeth, by Mr. FOX. N. B. These several Lectures are so arranged, that no two of them interfere in the hours of attendance; and the whole is calculated to form a complete Course of Medical and Chirurgical Instructions.—Terms and other Particulars may be learnt at the respective Hospitals.

THEATRE, LONDON HOSPITAL.

Dr. DENNISON and Dr. BYAM DENNISON will commence their Course of Lectures on the Theory and Practice of Midwifery and the Diseases of Women and Children, on Monday October the 2d, at Eleven o'Clock in the Forenoon.—For particulars apply at Dr. Dennison's, 37, Broad Street Buildings, or to Mr. Price, at the Hospital.

---

Early in October, Dr. ANDERSON will commence a Course of Lectures and Lessons in Chemistry and Pharmacy, at his House, 47, Frith Street, Soho.—The Lectures will contain the Institutes of Chemistry, illustrated by various and appropriate experiments; and in the Lessons, their practical application to the operations of Pharmacy will be shown and fully explained.—Fee to the Lectures and Lessons, Four Guineas.—Private instructions in Pharmacy.

Dr. BUXTON will, on the 2d of October, commence his Autumnal Course of Lectures on the Theory and Practice of Medicine, at the Medical Theatre, London Hospital.

Mr. CARPUE will commence his Lectures on Anatomy and Surgery, on the 1st of October.—Particulars may be known by applying to Mr. Carpue, No. 50, Dean Street, Soho.

Dr.

Dr. Clarke and Mr. Clarke will begin their Winter Course of Lectures on Midwifery, and the Diseases of Women and Children, on Thursday, October the 5th.—The Lectures are read every day at the House of Mr. Clarke, No. 10, Upper John Street, Golden Square, from a Quarter past Ten o'Clock in the Morning till a Quarter past Eleven, for the convenience of Students attending the Hospitals.—For particulars apply to Dr. Clarke, No. 1, New Burlington Street, or to Mr. Clarke, No. 10, Upper John Street, Golden Square.

Dr. Clough, Physician Man-midwife to the St. Mary-le-bone General Dispensary, &c. will commence his Winter Course of Lectures on Puerperal Anatomy, Physiology, and Pathology, on Monday, the 9th of October, at Ten in the Morning, at his House, No. 68, Berners Street; and (for the convenience of Students attending other classes) his Evening Course on the Tuesday following, at Seven o'Clock, and will be continued at the same hour on Tuesdays, Thursdays, and Saturdays, during the winter. In the course of these Lectures, Dr. Clough will have an opportunity of elucidating each subject by appropriate specimens of anatomical preparations; and of explaining difficult and laborious cases, by an interresting series of distorted pelves, from the first degree of triangular shape to that of the greatest on record, wherein the Cæsarian Section was performed; and the anatomy of the gravid uterus, by models formerly in the collection of Dr. Smellie and Mr. Cruickshank. These will render this part of the Course peculiarly interesting and valuable.—A Syllabus and Prospectus of which may be obtained as above; and at J. Callow's Bookseller, Crown Court, Soho.

Dr. Clutterbuck will begin his Autumn Course of Lectures on the Theory and Practice of Physic, Materia Medica, and Chemistry, on Wednesday October 4, at half-past Nine o'Clock in the Morning.—Further particulars may be known on application, at his House, No. 1, Crescent, New Bridge Street, Blackfriars.

Dr. Hooper will commence a Course of Lectures on the Theory and Practice of Physic, the Materia Medica, and Chemistry, on Monday, October the 23d, 1809, at Eight o'Clock in the Morning, at his Lecture Room, in Cork Street, Burlington Gardens.—Three Courses of the above Lectures will be delivered in the Year; which will commence in February, June, and October.

Dr. Reid will recommence his Course of Lectures on the Theory and Practice of Medicine, on Wednesday the 4th of October, at Nine o'Clock in the Morning, at his House, Grenville Street, Brunswick Square.—The subsequent Lectures will be delivered at the same place and hour, on every Monday, Wednesday, and Friday, until the conclusion of the Course.

Mr. Robertson will begin his next Course of Lectures on the Principles and Practice of Midwifery, and the Diseases of Women and Children, at Mr. Bell's Anatomical Museum, No. 10, Leicester Street, Leicester Square, on Friday, the 6th of October, at a quarter past Ten in the Morning.

Dr. SQUIRE, Ely Place, Holborn, will, on Tuesday the 2d of October, begin a Course of Lectures on the Theory and Practice of Midwifery, and the Diseases of Women and Children.

At the Theatre of Anatomy, Blenheim Street, Great Marlborough Street, Mr. BROOKES will commence his Autumnal Course of Lectures on Anatomy, Chemistry, and Surgery, on Monday the 2d of October, at Two o'Clock.

Mr. JOHN TAUNTON's Lectures on Anatomy, Physiology, Pathology, and Surgery, will commence on Saturday October the 7th, 1809, at Eight o'Clock in the Evening *precisely*, and be continued every Tuesday, Thursday, and Saturday, at the same hour.—Particulars may be had on applying to Mr. Taunton, Greville-Street, Hatton-Garden.

Mr. THOMAS's Lectures on the Principles and Operations of Surgery, will commence early in October, as usual. A Prospectus may be had at his House, Leicester Place, or at the Theatre of Anatomy in Windmill-Street.

Mr. WILSON will commence his Lectures on Anatomy, Physiology, Pathology, and Surgery, on Monday the 2d of October, at Two o'Clock, as usual, at his Theatre of Anatomy, Great Windmill Street.——A room is open for Practical Anatomy, under the direction of Mr. Wilson and Mr. Brodie, where demonstrations of the parts dissected are given daily from One until Two o'Clock. ——Mr. BRODIE will commence his Lectures on the Principles and Practice of Surgery in the beginning of October, as usual.

Dr. Saunders has published a fourth edition of his Treatise on the Structure, Economy, and Diseases of the Liver. This edition contains an Appendix, giving a more particular account of the Hepatitis of India, with observations on the prevalent use of mercury in the diseases of this country.

